# Evaluating homologous recombination activity in tissues to predict the risk of hereditary breast and ovarian cancer and olaparib sensitivity

**DOI:** 10.1038/s41598-024-57367-6

**Published:** 2024-04-08

**Authors:** Tokiwa Motonari, Yuki Yoshino, Moe Haruta, Shino Endo, Shota Sasaki, Minoru Miyashita, Hiroshi Tada, Gou Watanabe, Toshiro Kaneko, Takanori Ishida, Natsuko Chiba

**Affiliations:** 1 Breast and Endocrine Surgical Oncology, Tohoku University Graduate School of Medicine, 2-1, Seiryo-Machi, Aoba-Ku, Sendai, Miyagi 980-8575 Japan; 2 Department of Cancer Biology, Institute of Development, Aging and Cancer (IDAC), Tohoku University, 4-1 Seiryomachi Aoba-Ku, Sendai, Miyagi 980-8575 Japan; 3 Department of Cancer Biology, Tohoku University Graduate School of Medicine, 4-1 Seiryomachi Aoba-Ku, Sendai, Miyagi 980-8575 Japan; 4 Department of Electronic Engineering, Tohoku University, 6-6-05 Aoba Aramaki, Aoba-ku, Sendai, 980-8579 Japan; 5 Tohoku Medical and Pharmaceutical University, 1-15-1 Fukumuro, Miyagino-ku, Sendai, 983-8512 Japan

**Keywords:** Cancer, Biomarkers, Oncology, Risk factors

## Abstract

Homologous recombination (HR) repairs DNA damage including DNA double-stranded breaks and alterations in HR-related genes results in HR deficiency. Germline alteration of HR-related genes, such as *BRCA1* and *BRCA2*, causes hereditary breast and ovarian cancer (HBOC). Cancer cells with HR deficiency are sensitive to poly (ADP-ribose) polymerase (PARP) inhibitors and DNA-damaging agents. Thus, accurately evaluating HR activity is useful for diagnosing HBOC and predicting the therapeutic effects of anti-cancer agents. Previously, we developed an assay for site-specific HR activity (ASHRA) that can quantitatively evaluate HR activity and detect moderate HR deficiency. HR activity in cells measured by ASHRA correlates with sensitivity to the PARP inhibitor, olaparib. In this study, we applied ASHRA to lymphoblastoid cells and xenograft tumor tissues, which simulate peripheral blood lymphocytes and tumor tissues, respectively, as clinically available samples. We showed that ASHRA could be used to detect HR deficiency in lymphoblastoid cells derived from a *BRCA1* pathogenic variant carrier. Furthermore, ASHRA could quantitatively measure the HR activity in xenograft tumor tissues with HR activity that was gradually suppressed by inducible BRCA1 knockdown. The HR activity of xenograft tumor tissues quantitatively correlated with the effect of olaparib. Our data suggest that ASHRA could be a useful assay for diagnosing HBOC and predicting the efficacy of PARP inhibitors.

## Introduction

Homologous recombination (HR) contributes to the repair of DNA damage, including DNA double-stranded breaks (DSBs), inter-strand crosslinks, and stalled replication forks^1,2^. HR deficiency results in failure to repair DNA damage, which induces genomic instability and carcinogenesis^3^. Deleterious mutations or insufficient expression of HR-related genes results in HR deficiency. Germline alteration of the HR-related genes *BRCA1* and *BRCA2* cause hereditary breast and ovarian cancer (HBOC)^3^. Furthermore, a substantial proportion of spontaneous cancers in various organs contain mutations in HR-related genes^4^. Cancer cells with HR deficiency show increased sensitivity to cancer therapeutics including poly (ADP-ribose) polymerase (PARP) inhibitors, DNA-damaging agents, such as platinum reagents, and ionizing radiation; these agents create DNA damage that is repaired by HR^5,6^. Thus, evaluating HR activity is useful for diagnosing HBOC and predicting the effects of cancer therapeutics.

To diagnose HBOC, genetic tests using peripheral blood lymphocytes are performed to detect germline pathogenic variants of *BRCA1/2*, other HR-related genes, and other hereditary cancer-causing genes using multi-gene panels. Although the use of these genetic tests is well established, they class a substantial fraction of variants as variants of uncertain significance^7^ and they cannot detect HR deficiency caused by alterations in non-subject genes. Interestingly, previous reports showed that lymphocytes from germline *BRCA1/2* mutation carriers are radiosensitive^8,9^. This radiosensitivity might be caused by partial HR deficiency due to germline *BRCA1/2* mutation in one allele. Thus, detecting partial HR deficiency in somatic non-cancerous cells, including peripheral blood lymphocytes, could be useful for the diagnosis of HBOC.

The importance of detecting HR deficiency has increased following the clinical development of PARP inhibitors^10^. PARP inhibitors suppress the repair of DNA single-stranded breaks and trap PARP proteins at single-stranded break sites, resulting in DSBs and/or replication fork collapses^11^. HR-proficient cells can repair this DNA damage through HR, but not HR-deficient cells^11^. In this way, PARP inhibitors show synthetic lethality with HR deficiency. Thus, stratifying cancer patients by HR status is crucial to determine which will benefit from treatment with PARP inhibitors.

To stratify patients for treatment with PARP inhibitors, genetic tests of *BRCA1/2* and other HR-related genes are performed using peripheral blood lymphocytes and tumor tissues. However, similar to HBOC diagnosis, variants of uncertain significance are observed, even in *BRCA1/2,* and HR deficiency caused by alterations in non-subject genes is not detected. To overcome these limitations, genomic scar assays have been developed and used in clinical settings^12,13^. In these assays, various mutations caused by HR deficiency are detected by next-generation sequencing and the scar score is calculated from the burden of gene alterations. However, genomic scar assays cannot detect the restoration of HR activity by revertant mutations or other mechanisms. In addition, the scar score may increase regardless of HR activity because gene mutations accumulate with time. These limitations occur because gene alterations are used as a surrogate marker of HR activity. The direct measurement of HR activity in tumor cells may overcome these limitations.

**Figure 1:**
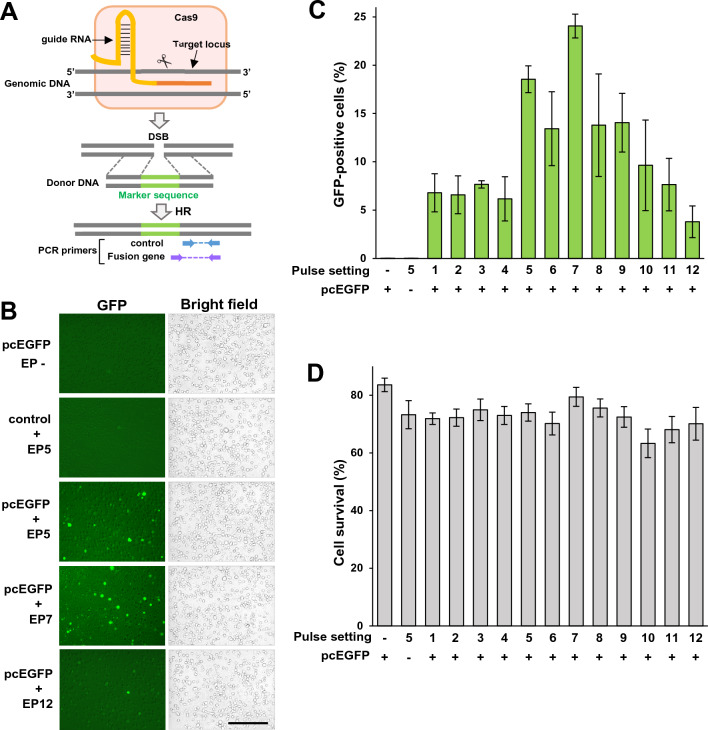
Plasmid delivery into lymphoblastoid cells by electroporation. (**A**) A schematic illustration of ASHRA. (**B**) Representative images of live HEV0012 cells. The pcEGFP plasmid was introduced into HEV0012 cells by electroporation using the indicated conditions in Table 1. The cells were analyzed after 72 h incubation. Scale bar, 100 μm. (**C**) The rate of GFP-positive cells measured by flow cytometry. The error bars indicate the standard error of the mean (SEM). (**D**) The cell survival cell rate (PI-negative fraction) by flow cytometry. The error bars indicate SEM.

We previously developed an assay, named assay for site-specific HR activity (ASHRA) to measure cellular HR activities (Fig. 1A)^14^. In ASHRA, the target gene is cut by Cas9 endonuclease. When a DSB is repaired by HR using the donor vector containing the marker sequence, the marker sequence is knocked into the target gene. The HR activity is then evaluated by measuring knock-in efficiency using quantitative PCR. ASHRA can detect HR deficiency by knockdown of HR-related genes, such as BRCA1 and RAD51^14^. We evaluated the HR activity of missense variants of BRCA1 and found that ASHRA can evaluate HR activity quantitatively and detect moderate HR deficiency that cannot be clearly discerned using the conventional direct repeat–green fluorescent protein (GFP) method^15–18^. HR activity measured by ASHRA was significantly correlated with sensitivity to the PARP inhibitor olaparib^18^. ASHRA can detect not only HR deficiency but also restoration of HR activity. In addition, because ASHRA does not require the establishment of stable cell lines required for the direct repeat-GFP method, ASHRA could be applied to a wide variety of patient samples. Thus, the next step will be to determine whether ASHRA can be used to estimate HR activity directly in patient-derived materials.

In this study, we optimized ASHRA protocols for use in lymphoblastoid cells and xenograft tumor tissues, which simulate peripheral blood lymphocytes and tumor tissues, respectively, as clinically available samples. We show that direct evaluation of HR activity could have potential for the diagnosis of hereditary cancer syndromes and stratification of patients for chemotherapy.

## Results

### Optimization of plasmid delivery by electroporation into lymphoblastoid cells

To evaluate HR activity by ASHRA, two plasmid vectors, a gRNA/Cas9 expression vector and a donor vector, need to be introduced into the live cells of interest (Fig. 1A)^14^. Previously, we used lipofection to deliver plasmids to adherent cells. However, lipofection has low efficiency for gene delivery into lymphocytes and cannot be used for tissues. In this study, we chose electroporation because it can introduce relatively large amounts of plasmids and is easy to perform.

Firstly, we optimized electroporation parameters for a lymphoblastoid cell line from a healthy donor, HEV0012, by transfecting a GFP expression vector. We systematically titrated the voltage, pulse duration, pulse repetition, and polarity switches (Table 1). The EP7 conditions showed the highest electroporation efficiency and tolerable cytotoxicity (Fig. 1B,C,D). We repeated optimization using several other normal lymphoblastoid cell lines and the EP7 conditions showed acceptable electroporation efficiency in these cell lines (data not shown).

**Table 1:** Pulse setting conditions used for electroporation.

Pulse setting	EP1	EP2	EP3	EP4	EP5	EP6	EP7	EP8	EP9	EP10	EP11	EP12
Voltage (V)	100				125				150			
Duration (msec)	5		2		5		2		5		2	
Repeats	2		6		2		6		2		6	
Polarity switch	−	+	−	+	−	+	−	+	−	+	−	+

### ASHRA detects HR deficiency in lymphoblastoid cells derived from a HBOC patient

**Figure 2:**
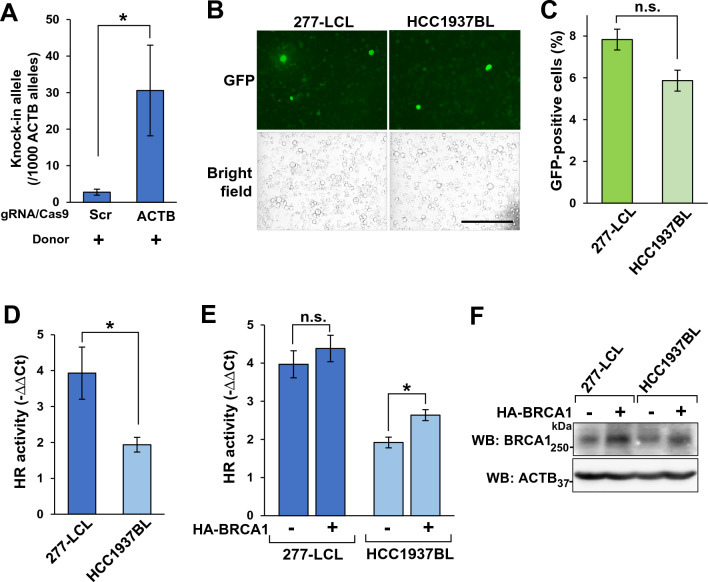
ASHRA detects HR deficiency in lymphoblastoid cells derived from a *BRCA1* carrier. (**A**) The efficiency of knock-in mediated by HR in HEV0012 cells. Plasmids for ASHRA were introduced by electroporation. After 72 h incubation, the genomic DNA was extracted and subjected to qPCR. The relative frequency of the knock-in alleles was calculated by the ΔCt method and is shown as the relative number per 1000 total *ACTB* alleles. The mean and SEM of three independent experiments are shown. *p < 0.05. (**B**) pcEGFP was introduced into the indicated cell lines under EP7 conditions. After 72 h incubation, representative images of live cells were taken. Scale bar, 100 μm. (**C**) The rate of GFP-positive cells in the samples used for panel (B) was analyzed by flow cytometry. The mean and SEM of three independent experiments are shown. n.s.: not significant. (**D**) The plasmid mixture was introduced into cells under EP7 conditions. After 72 h incubation, the relative amount of knock-in allele was measured by qPCR. The relative knock-in efficiency in each cell line relative to that in samples expressing scrambled gRNA is shown as ΔΔCt values. The mean and SEM of three independent experiments are shown. *p < 0.05. (**E**) The HA-BRCA1 expression vector or the empty vector was co-introduced into 277-LDL and HCC1937-BL cells with ASHRA plasmids by electroporation. The HR activities were measured as described in D. The mean and SEM of three independent experiments are shown. *p < 0.05, n.s.: not significant. (**F**) Whole-cell lysates of 277-LDL and HCC1937-BL cells after the introduction of the empty vector or the HA-BRCA1 expression vector were subjected to Western blotting.

Using the EP7 conditions, we introduced the two plasmid vectors for ASHRA into HEV0012 cells. The cells were cultured for 72 h after electroporation and the genomic DNA was extracted. The amounts of knock-in alleles and *ACTB* alleles were measured by qPCR and the frequency of the knock-in alleles per 1000 *ACTB* alleles was calculated (Fig. 2A). The knock-in allele frequency was significantly higher in the samples expressing the gRNA target gene *ACTB* than in the samples expressing non-target gRNA. The difference in the knock-in allele frequency between these two samples is thought to represent HR activity of the cells^14^. Here onwards, we refer to this difference simply as HR activity.

Then, we measured the HR activities of lymphoblastoid cells derived from a healthy donor and an HBOC patient, 277-LDL and HCC1937-BL cells, respectively. HCC1937-BL is derived from a *BRCA1* pathogenic variant carrier^19^. The germline variant *5382insC* results in a truncated protein lacking the carboxy-terminal region of BRCA1 and causing HR deficiency^17,20^. The efficiency of the vector introduction was not significantly different between the two cell lines (Fig. 2B,C). The HR activity was significantly lower in HCC1937-BL cells than in 277-LCL cells (Fig. 2D). To examine whether low HR activity in HCC1937-BL cells was due to *BRCA1* deficiency, we introduced a HA-tagged BRCA1 expression vector with the plasmids for ASHRA. The forced expression of BRCA1 significantly increased the HR activity in HCC1937-BL cells, but not in 277-LCL cells (Fig. 2E,F). These data suggest that ASHRA could detect HR deficiency in lymphoblastoid cells from a HBOC patient.

### Establishment of a protocol to measure HR activity in tumor tissues.

**Figure 3:**
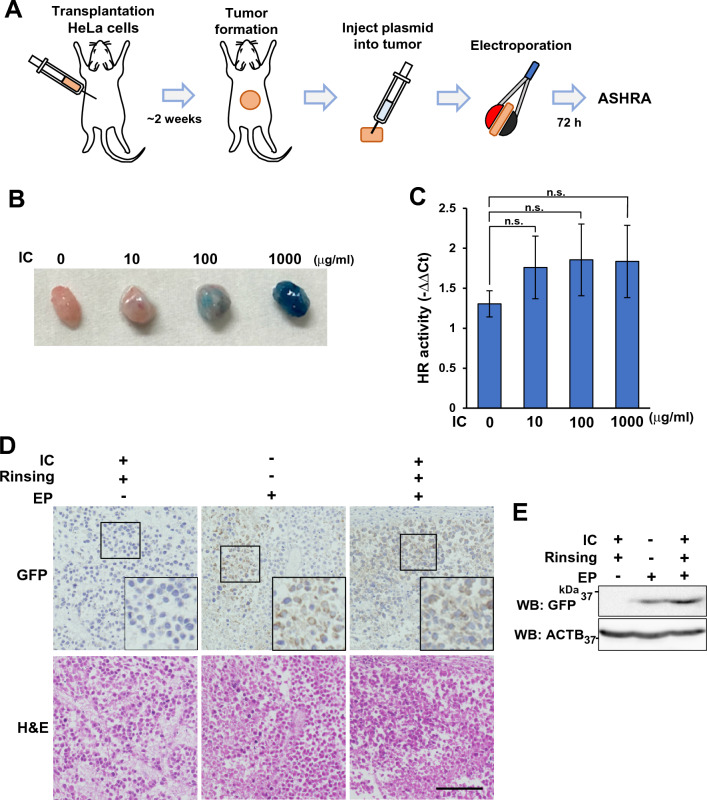
Plasmid delivery into xenograft tumor tissues by electroporation. (**A**) A schematic illustration of the procedure. (**B**) Images of tumors after injection of the plasmid solution with or without indigo carmine (IC: Indigo carmine). (**C**) The plasmids were introduced into tumor specimens using the indicated concentrations of IC. After 72 h incubation, the relative amount of knock-in allele was measured by qPCR. The knock-in efficiency in each cell sample relative to that in samples expressing scrambled gRNA is shown as ΔΔCt values. The mean and SEM of three independent experiments are shown. n.s.: not significant. (**D**) The pcEGFP vector was introduced into HeLa tumors as indicated. After 72 h incubation, the tumors were fixed and stained. Representative images are shown. H&E, Hematoxylin and eosin staining. Scale bar, 100 μm. (**E**) Whole-cell lysates of HeLa tumors electroporated with pcEGFP vector as indicated were analyzed by Western blotting.

Next, we attempted to measure the HR activity in tumor tissues. To establish a protocol for delivering plasmid vectors into tumor tissues, we injected the plasmid solution into a tumor specimen created by the subcutaneous transplantation of HeLa cells into nude mice (here onwards called a HeLa tumor) and applied electroporation pulses (Fig. 3A). To ensure the uniform and reproducible injection of the plasmid solution into the tumor tissue, we visualized the plasmid solution using indigo carmine. Indigo carmine is a biocompatible blue dye used to visualize mucosal lesions in gastroendoscopy, and does not bind nucleic acids and is not toxic to cells in in vitro culture. We injected plasmid vectors mixed with different concentrations of indigo carmine into HeLa tumor specimens and applied electroporation. After incubation of HeLa tumor specimens in DMEM for 72 h, we measured the HR activities by qPCR. The use of indigo carmine, regardless of its concentration, tended to improve the detection sensitivity (Fig. 3B,C). In subsequent experiments, we used 100 µg/ml indigo carmine in the plasmid solution.

In addition, we examined whether rinsing tumor tissues with 5% glucose solution to remove electrolytes before electroporation increased the detection of HR activity. Rinsing tended to increase the detection of HR activity, although the effect was not significant (data not shown).

Furthermore, we confirmed the introduction of plasmid vectors into tumor tissues by transfection of a GFP expression vector. After electroporation, HeLa tumors were incubated in DMEM for 72 h, and formalin-fixed, paraffin-embedded specimens of the tumor were prepared. As shown in Fig. 3D, GFP expression was observed in electroporated tissues and expression was more efficient in plasmid solutions that contained indigo carmine and were rinsed with 5% glucose solution before electroporation. GFP expression in the tumors was also confirmed by Western blotting (Fig. 3E). These results indicate that the vectors were successfully delivered into the tumor cells.

### Partial HR deficiency is induced in HeLa-tet-shBRCA1 cells

To examine whether the direct evaluation of HR activity in tumors is clinically useful, we investigated the correlation between HR activity in tumors and the sensitivity to the PARP inhibitor, olaparib. To control HR activity, we utilized HeLa-tet-shBRCA1 cells, in which shRNA expression against *BRCA1* is induced by tetracycline or doxycycline (Dox) treatment^21^.

**Figure 4:**
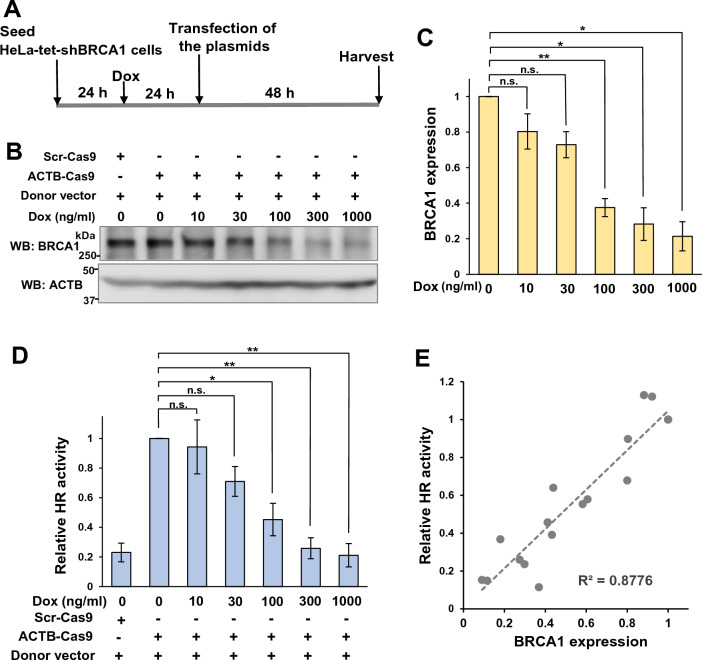
Gradual HR deficiency induced in HeLa-tet-shBRCA1 cells. (**A**) The protocol used for doxycycline (Dox) treatment of cell cultures. (**B**) Whole-cell lysates of HeLa-tet-shBRCA1 cells were subjected to Western blotting after the indicated Dox treatments. (**C**) BRCA1 expression in HeLa-tet-shBRCA1 cells treated with the indicated concentration of Dox. BRCA1 expression was determined by densitometry of Western blotting images. The mean and SEM of three independent experiments are shown. *p < 0.05, **p < 0.01, n.s.: not significant. (**D**) HR activity in HeLa-tet-shBRCA1 cells treated with the indicated concentration of Dox. HR activity was determined by ASHRA. The mean and SEM of three independent experiments are shown. *p < 0.05, **p < 0.01, n.s.: not significant. (**E**) The relationship between BRCA1 expression level and HR activity.

We treated HeLa-tet-shBRCA1 cells with various concentrations of Dox and analyzed the expression of BRCA1 protein (Fig. 4A). BRCA1 knockdown levels in HeLa-tet-shBRCA1 cells depended on the Dox concentration in the media (Fig. 4B,C ). Dox also decreased the HR activity measured by ASHRA in a concentration-dependent manner (Fig. 4D). Thus, the expression levels of BRCA1 correlated with HR activity (Fig. 4E).

### ASHRA quantitatively evaluates HR deficiency in tumor tissues

**Figure 5:**
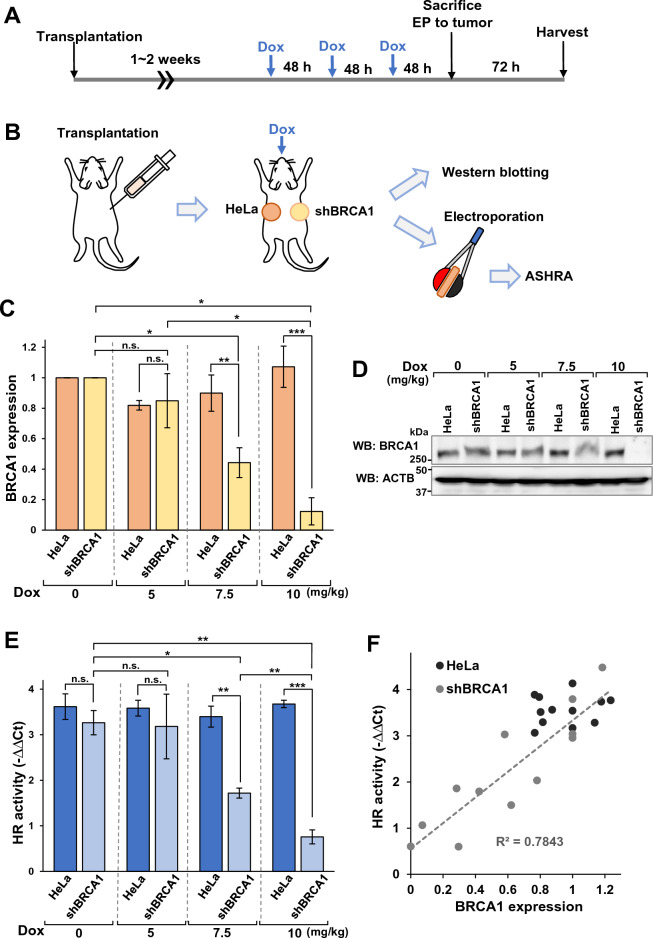
Evaluation of HR deficiency induced by BRCA1 knockdown in tumor tissues. (**A**, **B**) A schematic illustration of the BRCA1 knockdown procedure. (**C**, **D**) The tumor specimens excised after Dox administration were subjected to Western blotting. The density of bands in the blot was measured and the relative density of each condition was calculated. The mean relative density of the band of tumor samples from three individual mice with HeLa tumors (HeLa) or tet-shBRCA1 tumors (shBRCA1) is shown in (**C**). The mean and SEM of three independent experiments are shown. *p < 0.05, **p < 0.01, ***p < 0.001, n.s.: not significant. (**E**) The plasmids for ASHRA were introduced into the tumor specimens excised after Dox administration. After 72 h incubation, the samples were subjected to qPCR. The knock-in efficiency relative to that in samples expressing scrambled gRNA in each cell sample is shown as ΔΔCt values. The mean and SEM of three independent experiments are shown. *p < 0.05, **p < 0.01, ***p < 0.001, n.s.: not significant. (**F**) The BRCA1 expression shown in (C) and the HR activity shown in E were plotted. The broken line is the regression line of the data from HeLa-tet-shBRCA1 tumors.

To examine whether the extent of HR deficiency could be evaluated in tumor tissues by ASHRA, we created tumors by subcutaneously transplanting HeLa-tet-shBRCA1 cells in nude mice. After tumor formation, Dox was orally administered every other day and the tumor was excised from the mice after three doses of Dox (Fig. 5A,B). Total proteins were extracted from a piece of the tumor to evaluate BRCA1 expression by Western blotting (Fig. 5C,D ). BRCA1 expression was significantly knocked down by 10 mg/kg and 7.5 mg/kg Dox, and 10 mg/kg had a greater effect than 7.5 mg/kg Dox. In addition, 5 mg/kg of Dox slightly but not significantly knocked down BRCA1 expression. These data showed that the dose of Dox controlled the expression level of BRCA1 in HeLa-tet-shBRCA1 tumors in vivo.

Using the same tumor samples, we introduced the plasmids for ASHRA into the tumor tissues by electroporation and measured the HR activity by qPCR according to the protocol shown in Fig. 3. The HR activity of HeLa-tet-shBRCA1 tumors was significantly suppressed by 10 mg/kg and 7.5 mg/kg Dox, with 10 mg/kg having a greater effect (Fig. 5E). Administration of 5 mg/kg Dox did not suppress HR activity (Fig. 5E). HR activity in HeLa-tet-shBRCA1 tumors correlated with the BRCA1 expression controlled by Dox administration (Fig. 5F). On the other hand, Dox at all doses tested did not affect BRCA1 expression or HR activity in HeLa tumors (Fig. 5C–F). These data suggest that HR activity in HeLa-tet-shBRCA1 tumors could be controlled by Dox dose. For subsequent experiments, we regarded HeLa-tet-shBRCA1 tumors treated with 7.5 and 10 mg/kg Dox as having moderate and severe HR deficiency, respectively. We used 5 and 0 mg/kg Dox, which did not induce detectable HR deficiency, as controls.

### HR activity in tumor tissues measured by ASHRA correlates with the efficacy of olaparib

**Figure 6:**
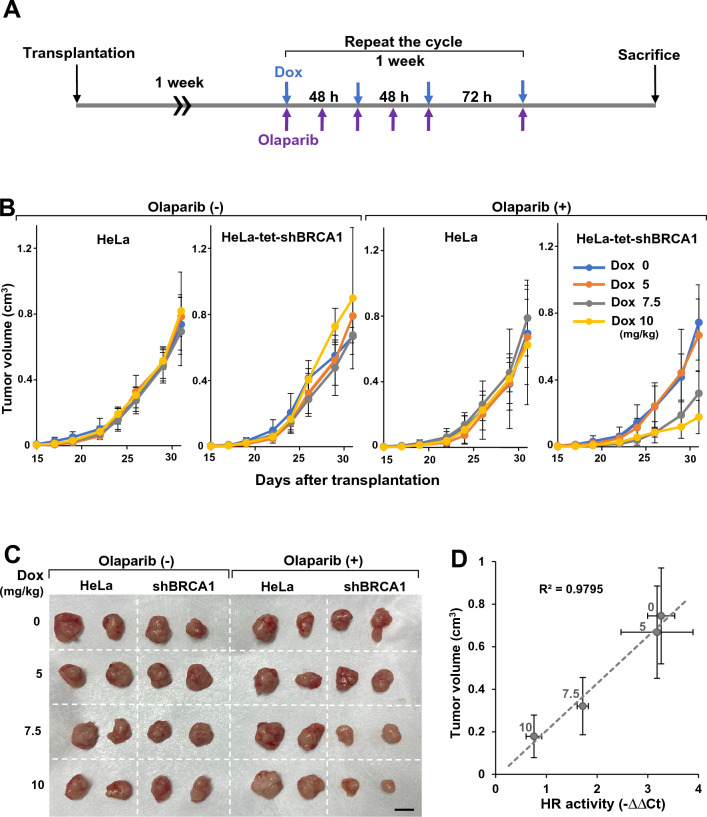
HR activity in tumor tissues correlated with olaparib efficacy. (**A**) A schematic illustration of the olaparib and Dox administration procedure. (**B**) The growth of each tumor under the indicated Dox treatment with or without olaparib administration. Data consist of three mice per treatment group. The error bars indicate the SD. No significant difference was detected between all Dox dose cohorts in HeLa tumors not treated with olaparib (*p* = 0.93), HeLa tumors treated with olaparib (*p* = 0.81), and HeLa-tet-shBRCA1 tumors not treated with olaparib (*p* = 0.66). For HeLa-tet-shBRCA1 tumors treated with olaparib, the *p*-values were as follows: *p* = 0.45 (Dox 0 vs. 5 mg/kg), *p* = 0.0002 (Dox 5 vs. 7.5 mg/kg),* p* < 0.0001 (Dox 5 vs. 10 mg/kg), and *p* = 0.44 (Dox 7.5 vs. 10 mg/kg). (**C**) Representative images of the tumors. Scale bar, 10 mm. (**D**) The correlation between HR deficiency and olaparib efficacy. The HR activity in Fig. 5E and the size of HeLa-tet-shBRCA1 tumors in mice administrated olaparib on day 31 and 0–10 mg/kg Dox were plotted. The numbers in the graph indicates the doses of Dox in mg/kg. The error bars indicate SD.

To evaluate the therapeutic efficacy of PARP inhibitors in tumors with various levels of HR deficiency in vivo, we orally administered olaparib with Dox to mice after transplantation of HeLa or HeLa-tet-shBRCA1 cells (Fig. 6A). To ensure the same treatment conditions, HeLa cells and HeLa-tet-shBRCA1 cells were transplanted on the left and right sides, respectively, of the same mouse. Olaparib (0 or 100 mg/kg) was administered once daily every weekday. Dox (0, 5, 7.5, or 10 mg/kg) was administered once a day, three times a week (i.e., on Monday, Wednesday, and Friday).

The volume of the HeLa-tet-shBRCA1 tumors was similar to that of HeLa tumors at all Dox doses without olaparib treatment (Fig. 6B,C ). In mice treated with olaparib, olaparib treatment did not affect the growth of HeLa tumors at any Dox dose (Fig. 6B,C ). Olaparib treatment did not significantly suppress the growth of HeLa-tet-shBRCA1 tumors treated with 0 and 5 mg/kg Dox (Fig. 6B,C). By contrast, olaparib significantly suppressed the growth of HeLa-tet-shBRCA1 tumors treated with medium and high-dose (7.5 and 10 mg/kg, respectively) Dox (Fig. 6B,C).

The anti-tumor efficacy of olaparib was concordant with the level of HR deficiency induced by Dox administration (Fig. 6D). Importantly, the correlation between HR activity and anti-tumor efficacy seemed to be quantitative. This finding suggests that the direct evaluation of HR activity in tumor using ASHRA might enable us to quantitatively predict the therapeutic efficacy of olaparib.

## Discussion

In this study, we successfully used ASHRA to measure the HR activity in lymphoblastoid cells and xenograft tumor tissues. ASHRA detected HR deficiency in lymphoblastoid cells derived from a HBOC carrier, and HR activity in xenograft tumor tissues correlated with the efficacy of olaparib.

To perform ASHRA, two plasmids must be efficiently delivered into live cells. In general, delivery of plasmid vectors into suspension cells or tissues is difficult, and several methods including electroporation, sonoporation, or gene gun systems, have been utilized^22,23^. In this study, we used electroporation to deliver the ASHRA plasmids into lymphoblastoid cells and xenograft tumor tissues.

The plasmid delivery efficiency for lymphoblastoid cells was dependent on the pulse conditions. For HEV0012 cells, EP7 conditions showed the highest delivery efficiency without significant cytotoxicity (Fig. 1C,D). Using the EP7 conditions, we obtained 8–25% GFP-positive cells for other lymphoblastic cell lines (data not shown). Although the best pulse condition might differ between cell lines, the EP7 conditions seem to enable enough plasmid delivery for ASHRA. For gene delivery to a tissue mass, we injected the plasmid solution into the tumor specimen and applied electroporation pulses. The use of a biocompatible dye, indigo carmine, enabled uniform injection of the plasmid solution into the tumor and tended to improve the delivery efficiency (Fig. 3B,C,D,E). Even at a very high concentration (1000 μg/ml), indigo carmine did not negatively affect the detection of HR activity (Fig. 3C). Collectively, our findings showed that ASHRA can be performed in lymphoblastoid cells or xenograft tumor tissues.

The amount of knock-in allele measured by qPCR was significantly lower in HCC1937-BL cells than in 277-LCL cells (Fig. 2D). The efficiency of knock-in depends not only on HR activity but also on other conditions including the efficiency of plasmid delivery. Therefore, we used 277-LCL cells that have a similar plasmid delivery efficiency as HCC1937-BL cells (Fig. 2B,C ). We showed that exogenously expressed BRCA1 increased the HR activity in HCC1937-BL cells, but not in 277-LCL cells. This finding suggests that BRCA1 expression in HCC1937-BL cells is insufficient for full HR activity despite the presence of one wild-type *BRCA1* allele, and is consistent with previous reports showing that lymphocytes from *BRCA1/2* mutation carriers are radiosensitive^8,9^. Overall, we showed that ASHRA could detect partial HR deficiency in lymphoblastoid cells from a *BRCA1* variant carrier. In this study, we analyzed HR activity only in one cell line, i.e., HCC1937-BL cells, derived from a HBOC patient due to the limited availability of other cell lines. Thus, an important future task is to verify our results using samples obtained from other HBOC patients. If we can detect partial HR deficiency in normal tissues from individuals that have pathogenic alterations in HR-related genes, such as *BRCA1* and *BRCA2*, we will be able to screen HBOC carriers using ASHRA regardless of the causative genes, and even if the causative genes are not known.

In this study, we used a lymphoblastoid cell line immortalized by Epstein-Barr virus. Although we found no previous reports noticing the effect of Epstein-Barr virus infection on HR activity, this possibility cannot be ruled out. As far as we know, plasmid delivery by electroporation is not generally utilized for primary non-immortalized peripheral blood cells. Thus, an important question is whether the ASHRA protocol can be further modified to measure HR activity in non-immortalized peripheral blood lymphocytes.

To investigate the correlation between HR activity measured by ASHRA and the efficacy of PARP inhibitors, it is necessary to analyze tumors with various HR statuses. Using panels of multiple cell lines was one of the options considered for the current study. However, HR activity determined by ASHRA, which measures the knock-in efficiency of a marker sequence, could be affected by the plasmid delivery efficiency, expression level of Cas9, and cell-cycle distribution, which vary among cell lines. To avoid these complexities, we utilized a Dox-inducible shRNA expression system. BRCA1 expression was suppressed by Dox in a concentration-dependent manner (Fig. 4C). Similarly, the degree of HR deficiency correlated with Dox concentration (Fig. 4D). Therefore, the BRCA1 expression level quantitatively correlated with the HR activity (Fig. 4E).

Using the HeLa-tet-shBRCA1 cell line, we showed that HR activity in tumor tissues correlated with the therapeutic efficacy of olaparib in vivo (Figs. 5,6). As expected, the tumors with severe HR deficiency induced by high-dose Dox responded markedly to olaparib, and the HR-proficient tumors did not significantly respond to olaparib (Fig. 6). Importantly, the tumors with moderate HR deficiency induced by medium-dose Dox responded to olaparib, but the response was less than that of tumors with severe HR deficiency (Fig. 6). These results suggest that the response of a tumor to olaparib can be quantitatively predicted by the degree of HR deficiency in the tumors.

In this study, we used only one cell line and its derivative cell line, HeLa and HeLa-tet-shBRCA1. However, we successfully measured HR activity in various other cells, including MCF7 cells^18^. Therefore, ASHRA could be applied to other types of cells and tissues. However, the efficiency of plasmid delivery varies between cell types and may affect ASHRA values. Therefore, procedures that normalize ASHRA values between samples should be developed in future studies.

At present, the clinical use of PARP inhibitors is determined by qualitative test results. However, we and other groups have shown that some variants of BRCA1 have the moderate HR deficiency and sensitivity to olaparib, and present an intermediate cancer risk^18,24–27^. It is difficult to manage individuals who have those variants during clinical decision making. For example, some cancers diagnosed as HR deficiency according to current diagnostic systems may actually have moderate HR deficiency and not respond well to standard doses of PARP inhibitors. Conversely, some cancers diagnosed as HR-proficient may actually have moderate HR deficiency. These cases may respond partially to PARP inhibitors and benefit from dose escalation.

In this study, we have shown that ASHRA can be used to measure directly HR activity in lymphoblastoid cells and xenograft tumors, which simulate clinically available specimens, suggesting that ASHRA could be a useful assay for evaluating HR deficiency in the clinic. In the future, quantitative diagnosis of HR deficiency in peripheral blood lymphocytes and tumors by ASHRA may help develop more precise treatments.

## Methods

### Cell lines

HeLa cells were obtained from the American Type Culture Collection (Manasas, VA, USA). HeLa-tet-shBRCA1 cells were established by stably integrating a tetracycline-inducible shRNA expression cassette into HeLa cells^21^. The human lymphoblastoid cell lines HEV0012 and 277-LCL were obtained from the Riken BioResource Research Center (Tsukuba, Japan) and the Cell Resource Center for Biomedical Research at Tohoku University (Sendai, Japan), respectively. The human lymphoblastoid cell line HCC1937-BL from a *BRCA1* pathogenic variant carrier was obtained from the American Type Culture Collection. HeLa cells were maintained in DMEM (Fujifilm-Wako Pure Chemicals, Osaka, Japan) supplemented with 8% fetal bovine serum (FBS, BioWest, Nuaille, France). HeLa-tet-shBRCA1 cells were maintained DMEM supplemented with 8% FBS and 1 μg/ml puromycin (Fujifilm-Wako Pure Chemicals). Human lymphoblastoid cell lines were maintained in RPMI1640 (Fujifilm-Wako Pure Chemicals) supplemented with 20% FBS. Cells were incubated at 37 °C in an atmosphere with 5% CO_2_.

### Evaluation of electroporation efficiency in lymphoblastoid cells

The enhanced GFP expression vector pcEGFP was constructed by cloning of the GFP coding sequence from pEGFP (Clontech, Mountain View, CA, USA) into pcDNA3.1. pcEGFP was introduced into cells by electroporation. After 72 h incubation, cells were suspended in DMEM containing 2 μg/ml propidium iodide (Fujifilm-Wako Pure Chemicals) and analyzed using a Cytomics FC500 flow cytometer (Beckman-Coulter, Brea, CA, USA).

### Measurement of HR activity in lymphoblastoid cells

Lymphoblastoid cells were suspended in Opti-MEM (Thermo Fisher, Waltham, MA, USA) at a density of 1 × 10^6^ cells/ml. Then, 10 μl of ASHRA assay plasmid solution (1.0 mg/ml, donor vector: gRNA/Cas9 expression vector = 2 : 1 in molar ratio) was added to 90 μl of the cell suspension and electroporation was applied. Donor vector (pBS-ACTB-C200-GFPfr1, Addgene ID: 169799) and gRNA/Cas9 expression vectors (LentiCRISPRv2-scr, Addgene ID: 169795 and LentiCRISPRv2-ACTB-C1, Addgene ID: 169796) have been described previously^14^. For electroporation, a NEPA21 electroporator and 2 mm gap cuvettes (Nepagene, Chiba, Japan) were used. Electroporation conditions are shown in Table 1. Cells were diluted with RPMI1640 containing 20% FBS immediately after electroporation and incubated for 72 h. Genomic DNA was extracted using a blood genomic DNA extraction mini kit (Favorgen, Ping Tung, Taiwan). Quantitative PCR was performed on a CFX96 Touch real-time PCR detection system (Bio-Rad, Hercules, CA, USA) using GoTaq qPCR master mix (Promega, Madison, WI, USA). Quantification of the knocked-in allele and control allele by qPCR was performed using the following primer sets: 5'-GTCCTGCTGGAGTTCGTGACCG-3' and 5'-GTGCAATCAAAGTCCTCGGC-3' for the knocked-in allele, and 5'-AGTTGCGTTACACCCTTTCTTG-3' and 5'-GTGCAATCAAAGTCCTCGGC-3' for the control allele. The relative quantity of the knocked-in allele was calculated by the ΔΔCt method.

### Western blotting

Whole-cell lysates of culture cells were prepared in 1 × sample buffer (50 mM Tris–HCl pH 6.8, 2% sodium dodecyl sulfate (SDS), 0.67 M 2-mercaptoethanol, 12% glycerol, and 1% bromophenol blue), sonicated, and incubated at 95 °C for 5 min. Tumor tissues were homogenized in T-PER reagent (Thermo Fischer Scientific) using a Biomasher II homogenizer (Nippi, Tokyo, Japan). After centrifugation at 20,000 *g* for 10 min, a half volume of 3 × Laemmli sample buffer was added to the supernatant. SDS-PAGE and Western blotting were carried out according to the conventional method with minor modifications. The following antibodies were used: a polyclonal anti-BRCA1 antibody specific for residues 1528–1863 of BRCA1, an anti-β-actin antibody (sc-47778 HRP, Santa Cruz Biotechnologies, Dallas, TX, USA), and anti-GFP antibody (Fujifilm-Wako Pure Chemicals). Uncropped images of Western blot are shown in Supplementary Fig. S1–8.

### Xenograft tumor formation in mice

HeLa (2.5 × 10^6^ cells) or HeLa-tet-shBRCA1 (4 × 10^6^ cells) cells were subcutaneously transplanted in male nude mice (BALBc nu/nu, CLEA Japan, Tokyo, Japan). Mice were euthanized by cervical dislocation. The mouse was sacrificed when the tumor diameter reached about 1.5 cm. All animal experiments were performed in accordance with a protocol approved by the Committee on Animal Experimentation of Tohoku University. All animal experiments were approved by the Committee on Animal Experimentation of Tohoku University. The animal study presented here is reported in accordance with ARRIVE guidelines.

### Measurement of HR activity in tumor tissues

The tumor mass was surgically excised from the mouse and trimmed to get 5–8 mm cubic specimens. ASHRA plasmid solution (total DNA amount 100 μg, 5 mg/ml, gRNA/Cas9 expression vector : donor vector = 1 : 2 in molar ratio) was manually injected into the tumor specimen using a 1 ml syringe with a 29G needle (Terumo Tokyo, Japan). After plasmid injection, the tumor specimen was rinsed with 5% glucose solution to remove electrolytes on the tumor surface and electroporation was applied to the specimen. For electroporation, a NEPA21 electroporator (Nepagene) and a forceps-type electrode (CUY650P7, Nepagene) were used. The specimen was incubated in DMEM containing 8% FBS for 72 h. The procedures used for extraction of genomic DNA, quantification of the knock-in allele, and measurement of HR activity were the same as those used for lymphoblastoid cells.

### Immunostaining

The pcEGFP vector was introduced into HeLa tumors. After 72 h incubation, the tumors were fixed in 10% formalin and embedded in paraffin. For immunostaining of GFP, slices of paraffin-embedded specimens were de-paraffinized in serial solutions of xylene and ethanol. The specimens were blocked with 1% BlockAce (KAC, Hyogo, Japan) in phosphate-buffered saline (PBS) for 1 h at room temperature. After blocking, the specimens were incubated with anti-GFP antibody (raised against the QSALSKDPNEKRDHM peptide in rabbits) diluted in PBS containing 0.5% bovine serum albumin overnight at 4 °C. After washing with PBS, endogenous peroxidase activity was removed by incubation in methanol with 0.03% H_2_O_2_ for 20 min at room temperature. For the secondary antibody, Envision + System HRP labelled polymer anti-rabbit (K4002, DAKO, Santa Clara, CA, USA) was used according to the manufacturers’ instructions. Hematoxylin–eosin staining was performed using standard methods by the Pathology Section of the Biomedical Research Core of Tohoku University Graduate School of Medicine.

### Drug treatment of mice

A stock solution of Dox (Fujifilm-Wako Pure Chemicals) was prepared in distilled water at 50 mg/ml. A stock solution of olaparib was prepared in Dimethyl sulfoxide at 25 mg/ml. For Dox administration, the required amount of stock solution was diluted in distilled water to 100 μl/mouse at the time of use and was orally administered using a feeding needle (FG5202, As One, Tokyo, Japan). For co-administration of Dox and olaparib, the Dox stock solution, the olaparib stock solution and 40 μl of PEG300 were mixed. The drug mixture was diluted with distilled water to 200 μl/mouse before administration. The administration schedules are indicated in Figs. 5A,6A.

### Statistical analysis

Statistical analysis was performed using JMP 14 software (SAS Institute Inc, Cary, NC, USA). Graphs were constructed using Excel 2016 (Microsoft, Redmond, WA, USA). A two-tailed Welch’s t-test was used to compare the means of two different samples. For comparison of tumor growth rate in vivo, generalized linear regression analysis was used. A *p*-value of < 0.05 was considered significant.

### Supplementary Information

Supplementary Information.

## Data Availability

The datasets generated during the current study are available from the corresponding authors on reasonable request.
