# Supplementary material for: Evaluating homologous recombination activity in tissues to predict the risk of hereditary breast and ovarian cancer and olaparib sensitivity
Source: Sci Rep. 2024 Apr 8;14:7519. doi: 10.1038/s41598-024-57367-6 (PMC11001962; doi:10.1038/s41598-024-57367-6)

Evaluating homologous recombination activity in tissues to predict the risk of hereditary breast and ovarian cancer and olaparib sensitivity

Tokiwa Motonari, Yuki Yoshino, Moe Haruta, Shino Endo, Shota Sasaki, Minoru Miyashita, Hiroshi Tada, Gou Watanabe, Toshiro Kaneko, Takanori Ishida, Natsuko Chiba

Figure S1

Figure 2F

WB: BRCA1

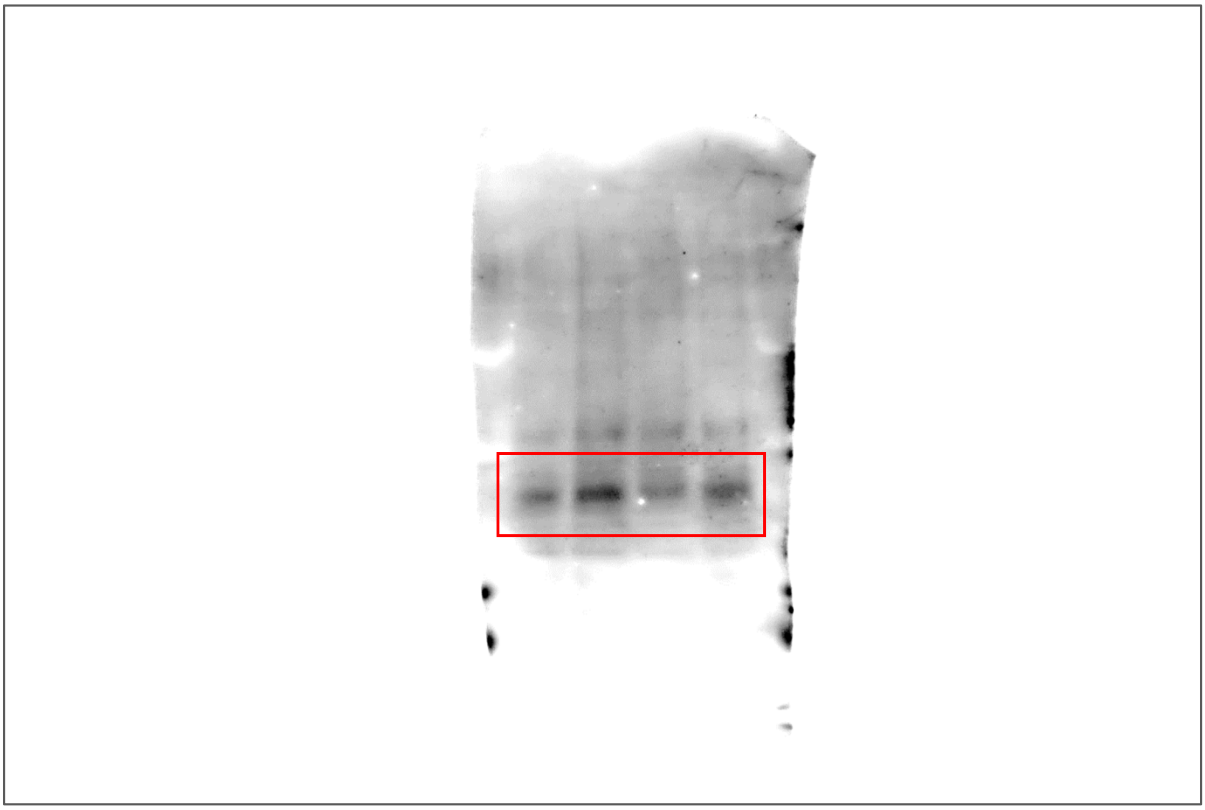

WB: BRCA1 (long exposure)

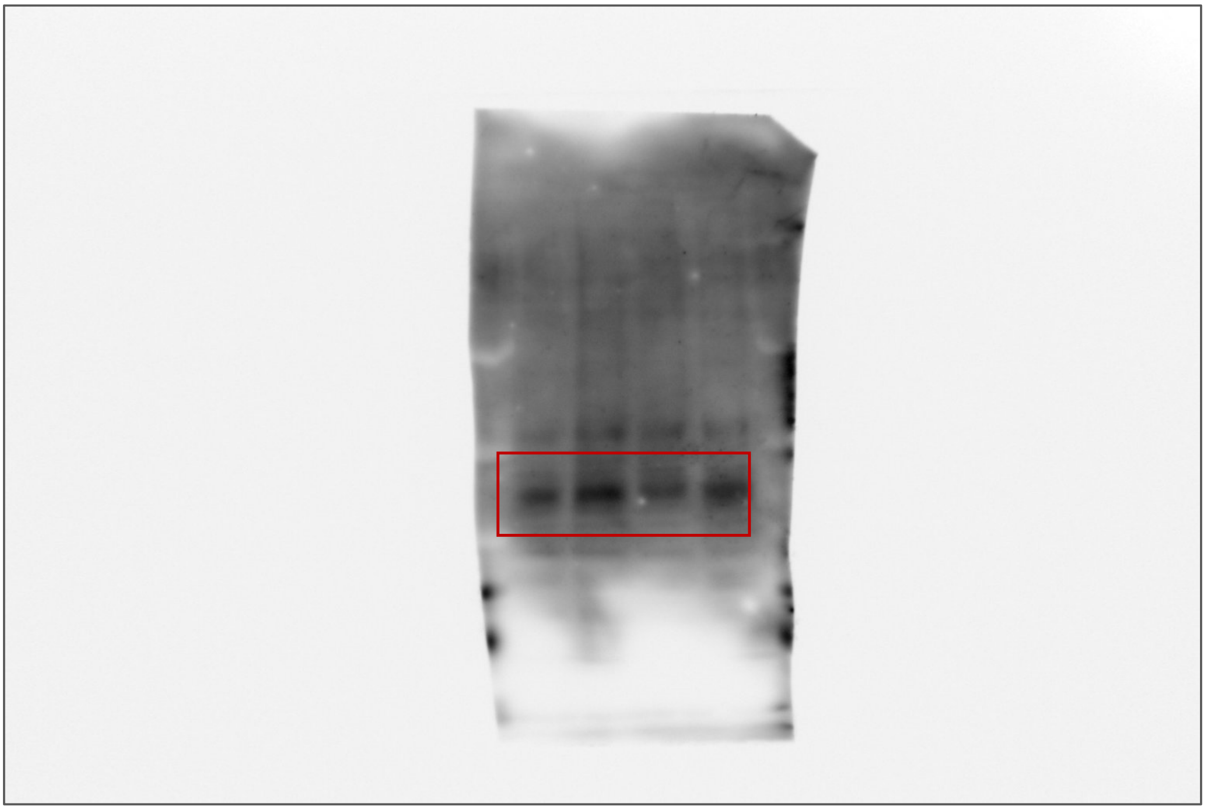

Figure S2

Figure 2F

WB: ACTB

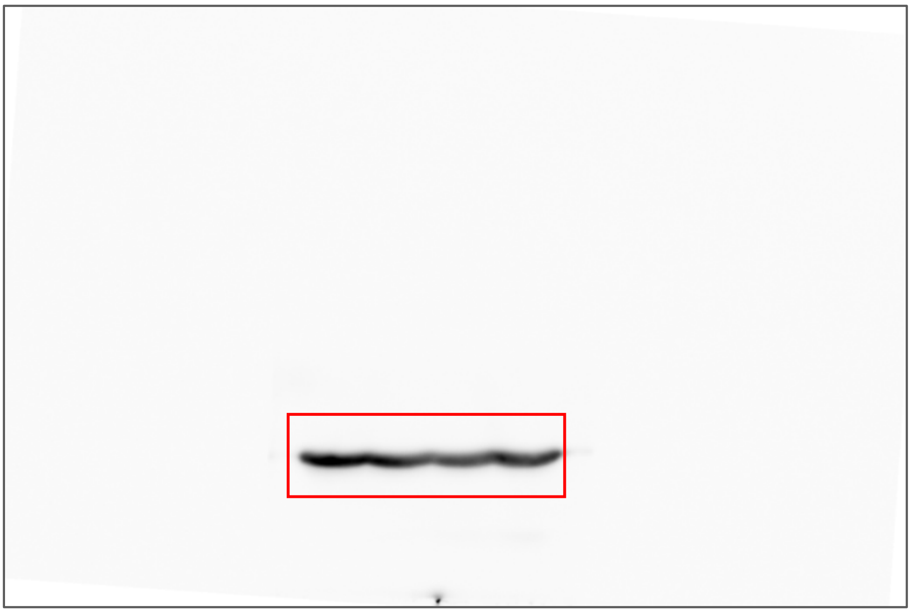

WB: ACTB (long exposure)

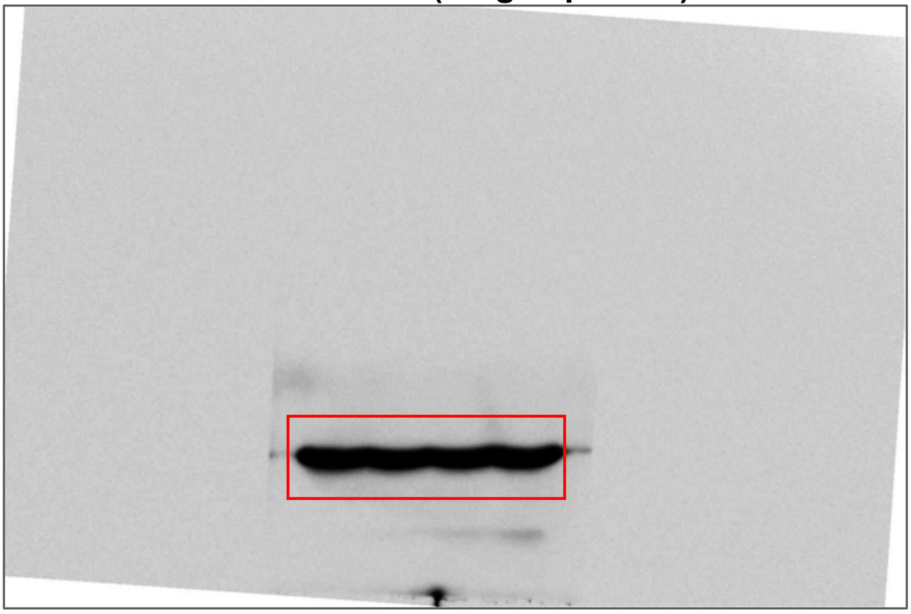

WB: ACTB (high contrast image of long exposure)

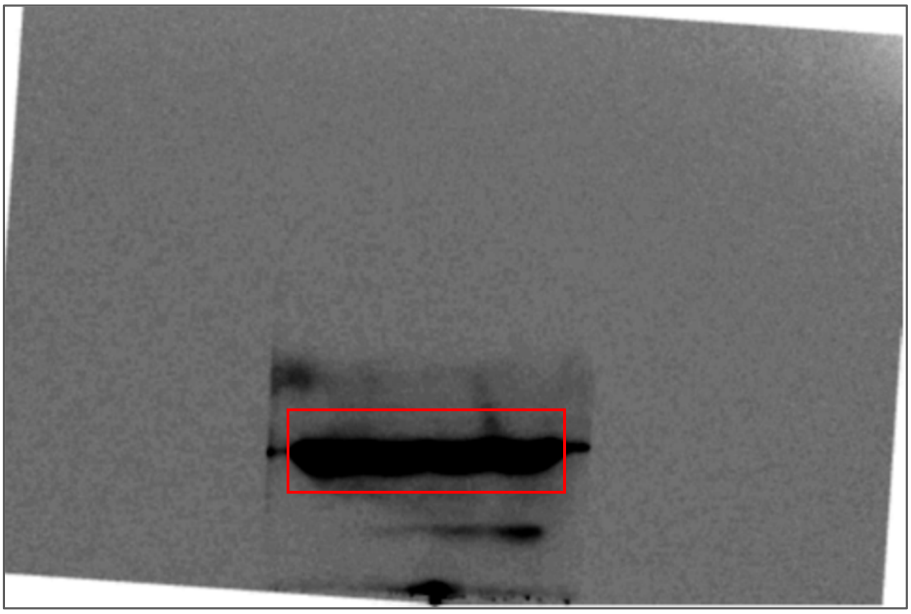

Figure S3

Figure 3E

WB: GFP

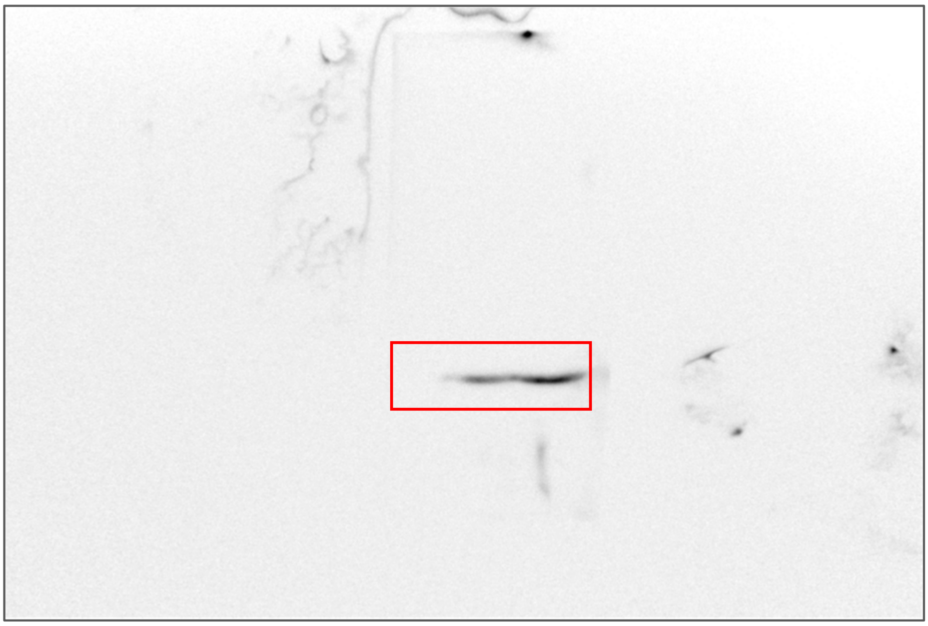

WB: GFP (long exposure)

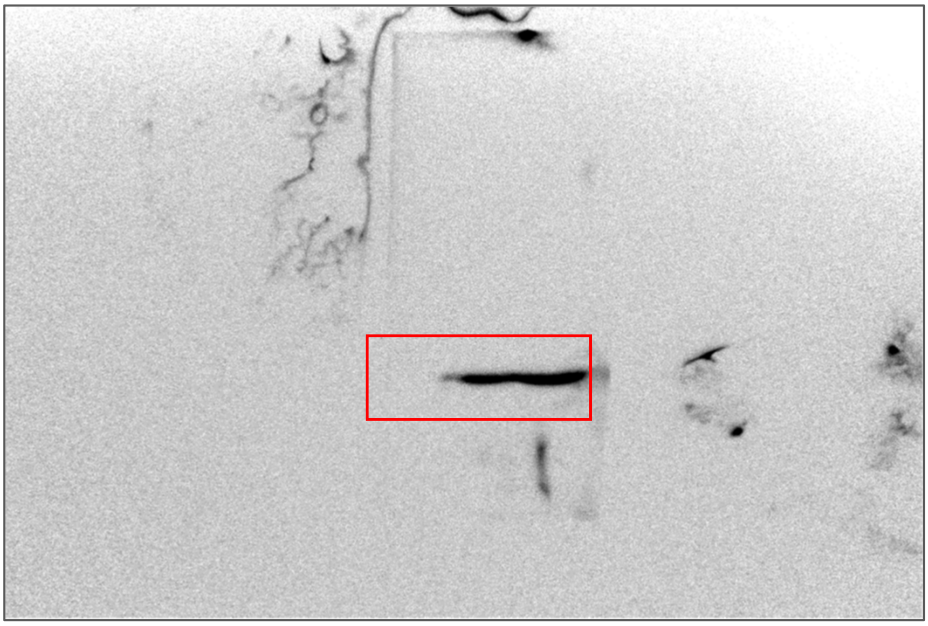

WB: GFP (high contrast image of long exposure)

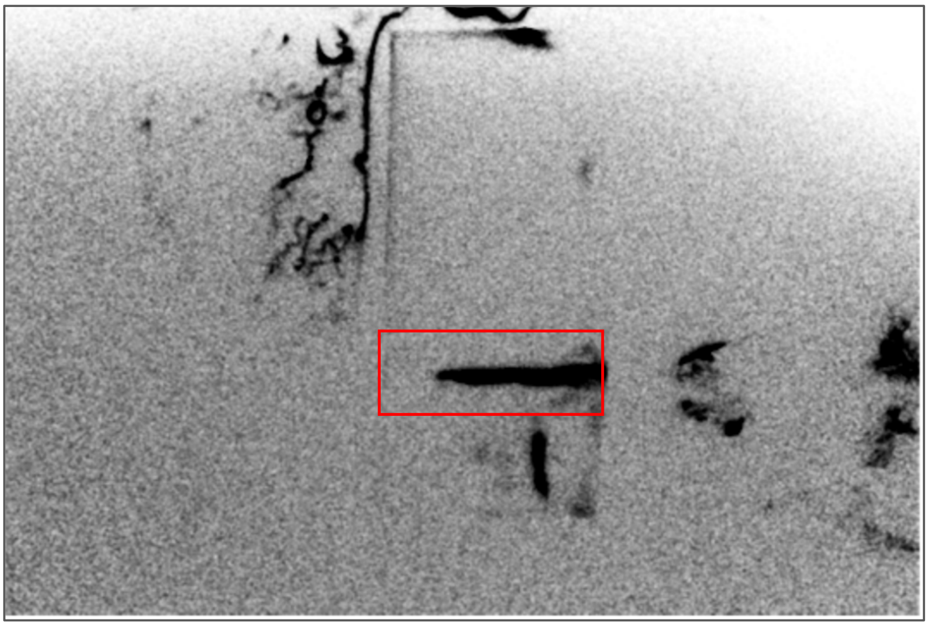

Figure S4

Figure 3E

WB: ACTB

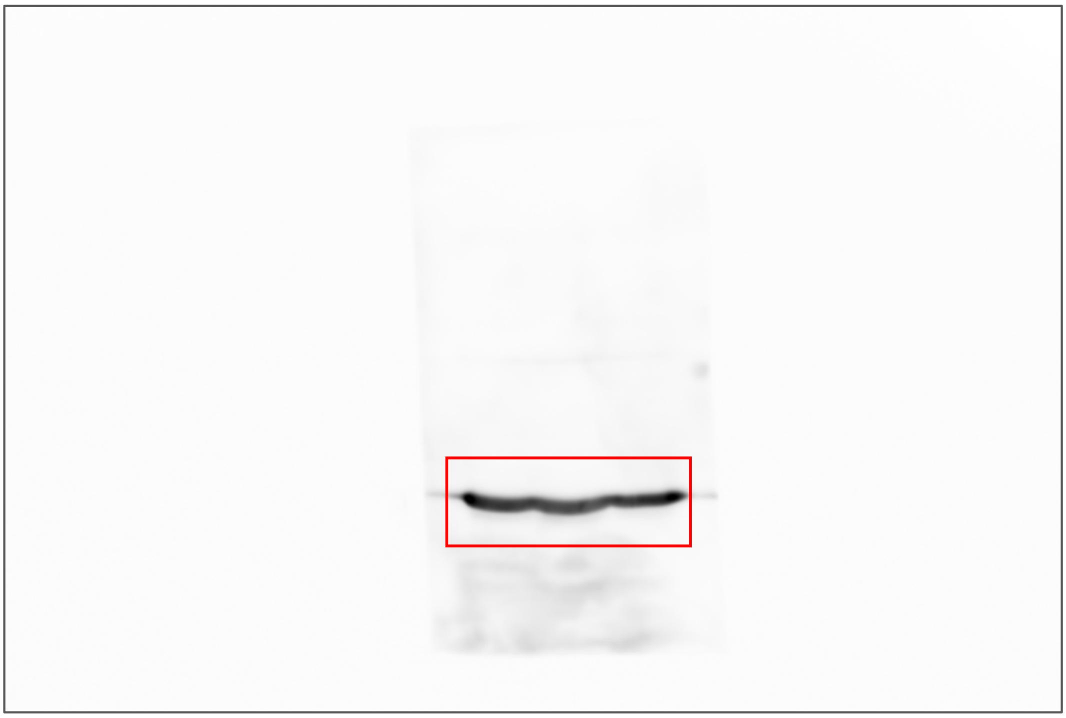

WB: ACTB (long exposure)

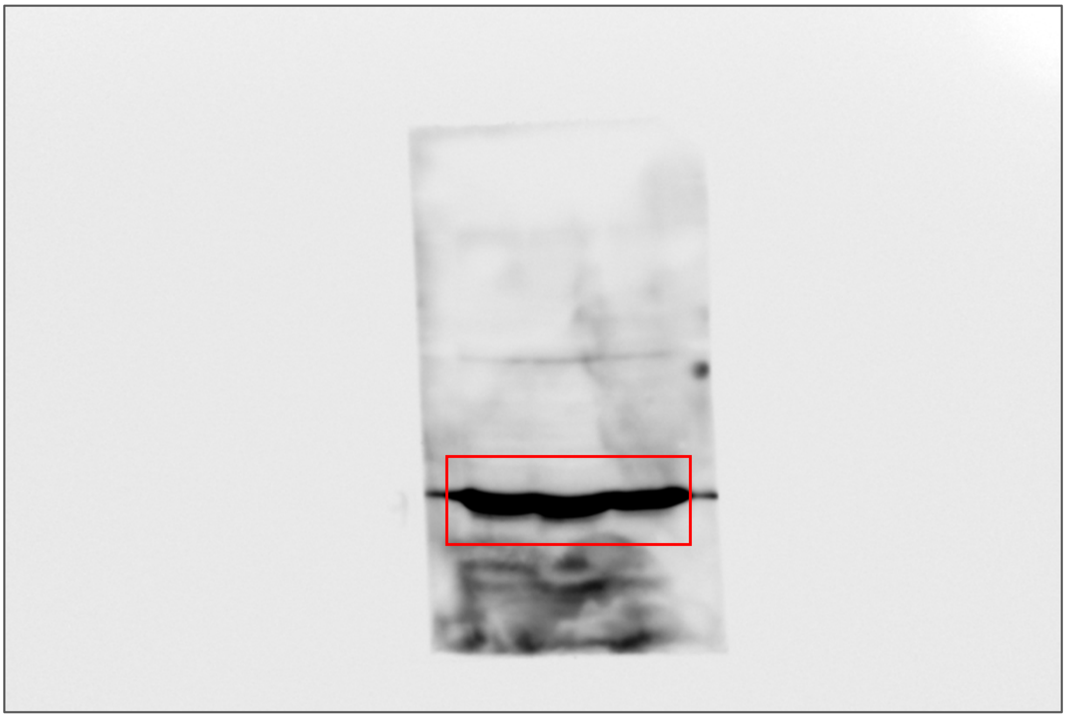

Figure S5

Figure 4B

WB: BRCA1

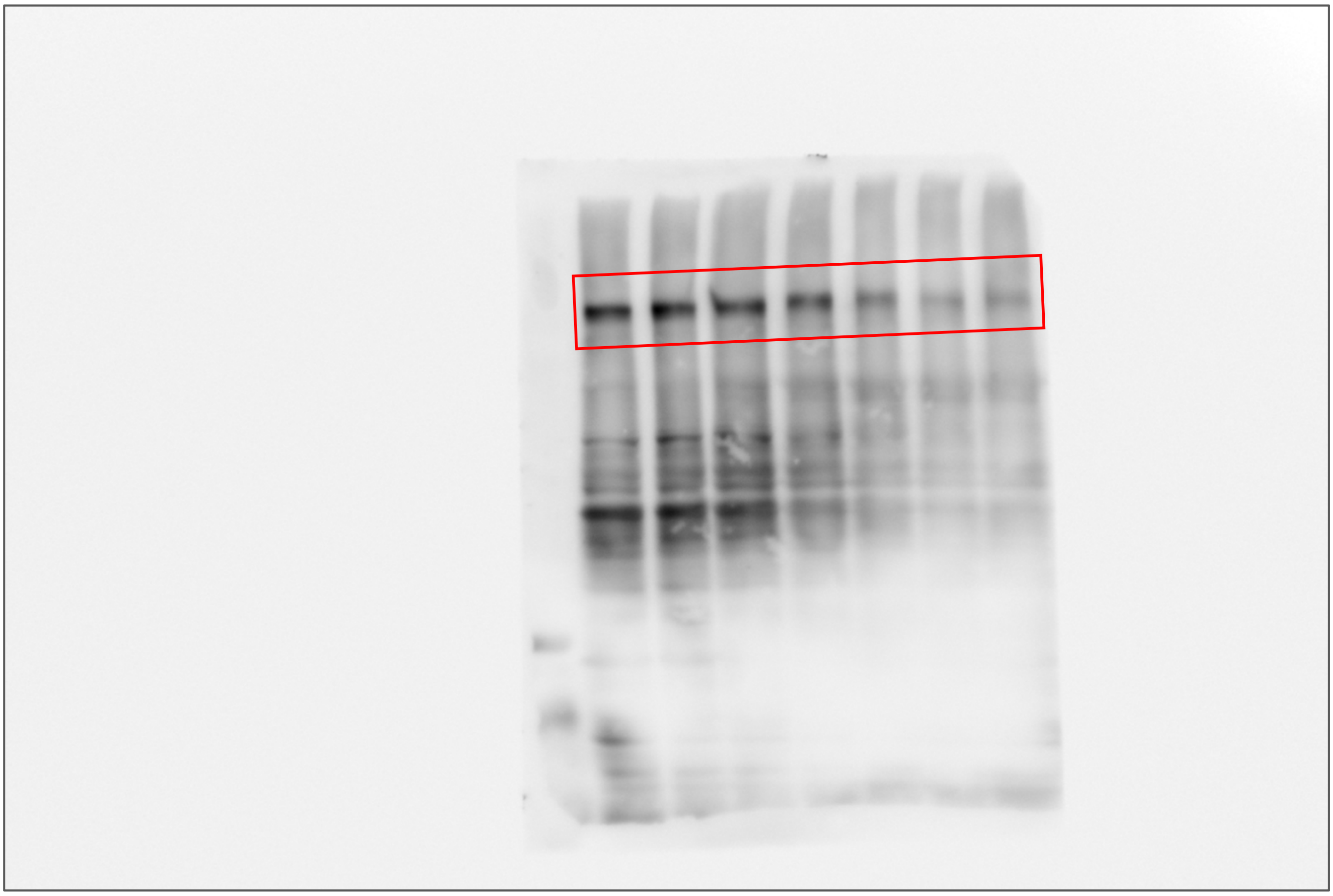

WB: BRCA1 (long exposure)

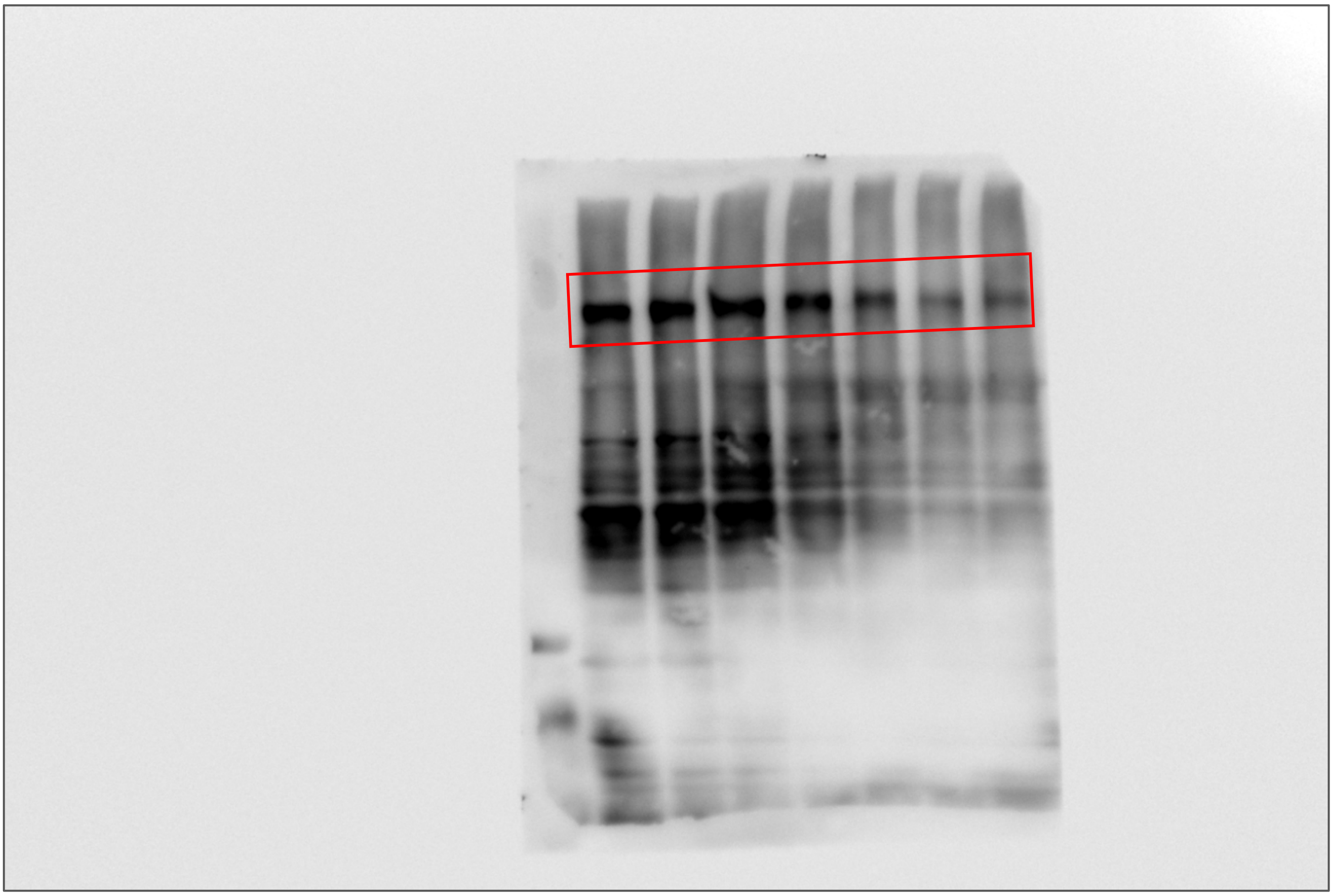

Figure S6

Figure 4B

WB: ACTB

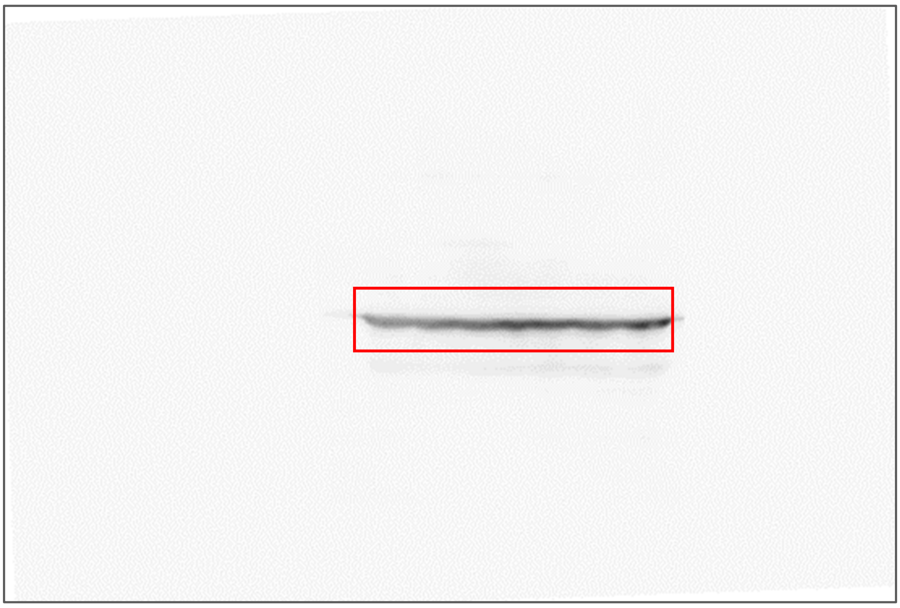

WB: ACTB (long exposure)

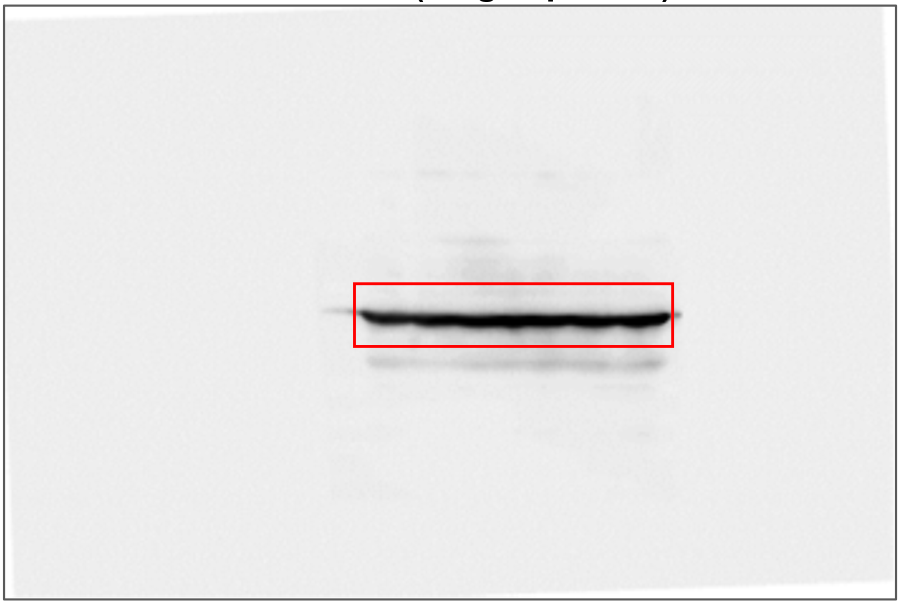

WB: ACTB (high contrast image of long exposure)

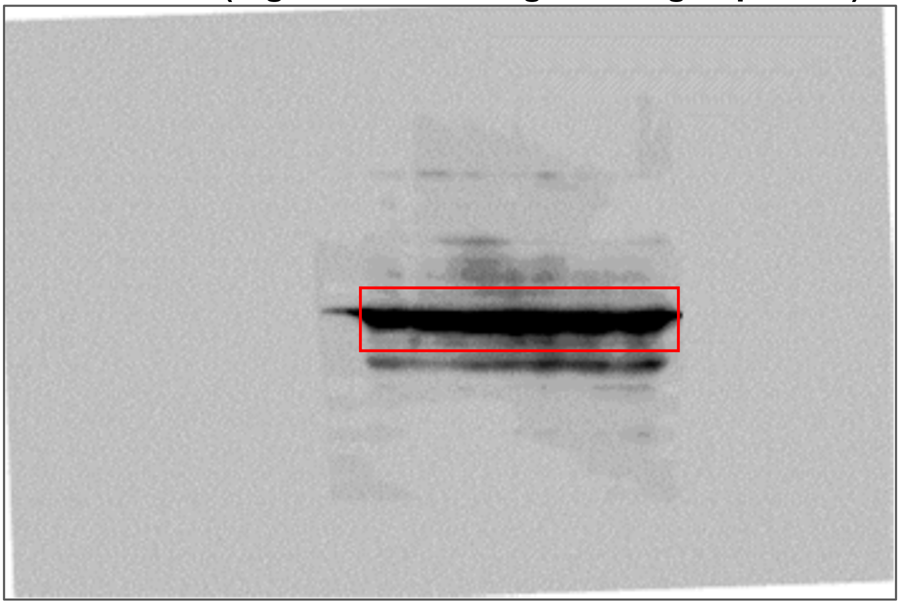

Figure S7

Figure 5D

WB: BRCA1

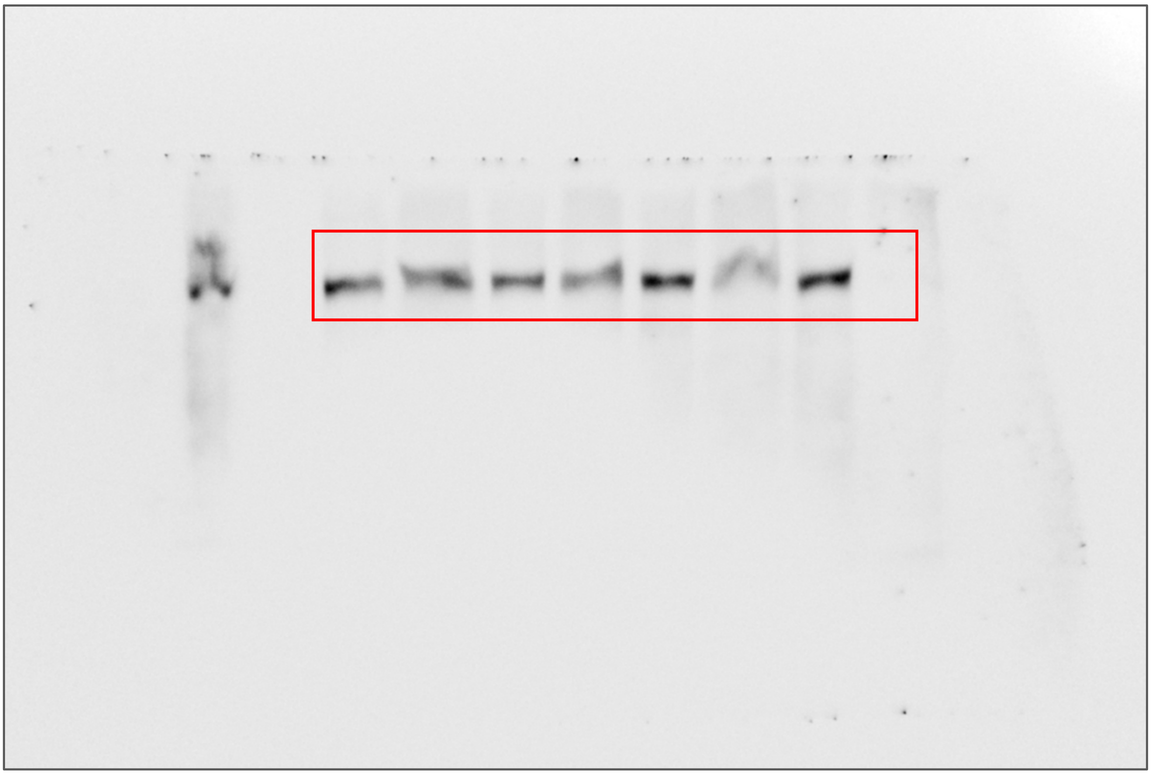

WB: BRCA1 (long exposure)

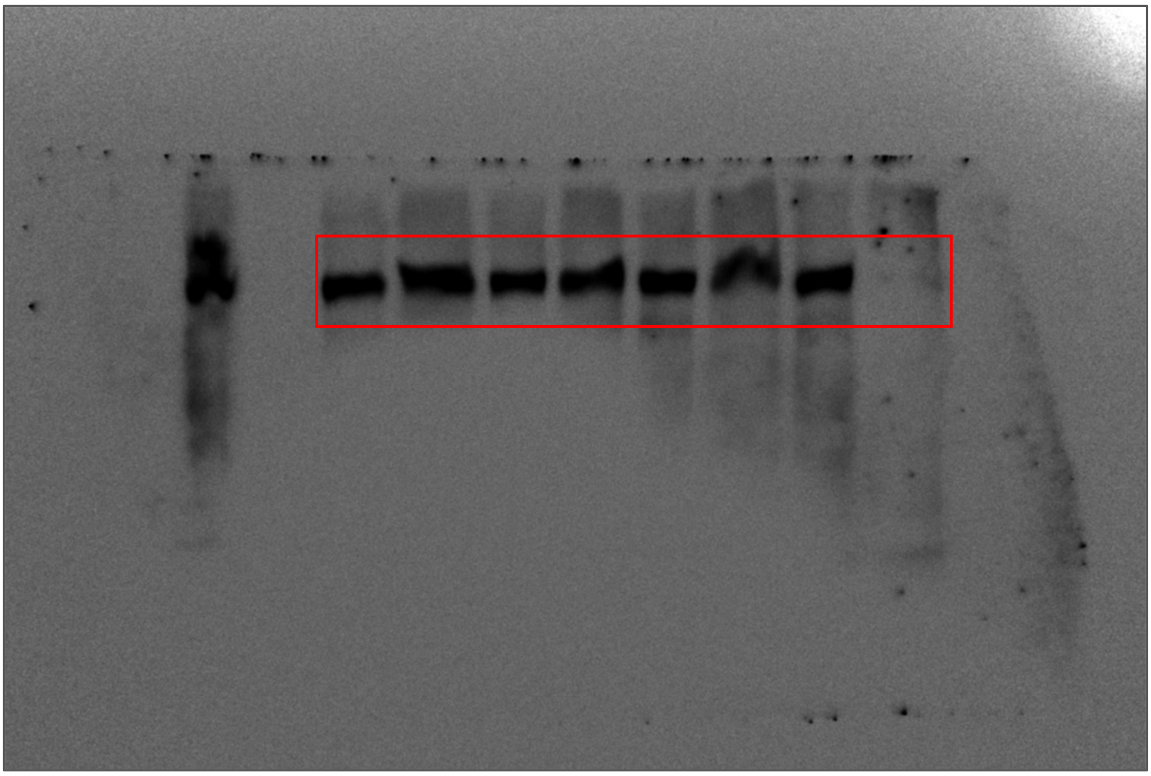

Figure S8

Figure 5D

WB: ACTB

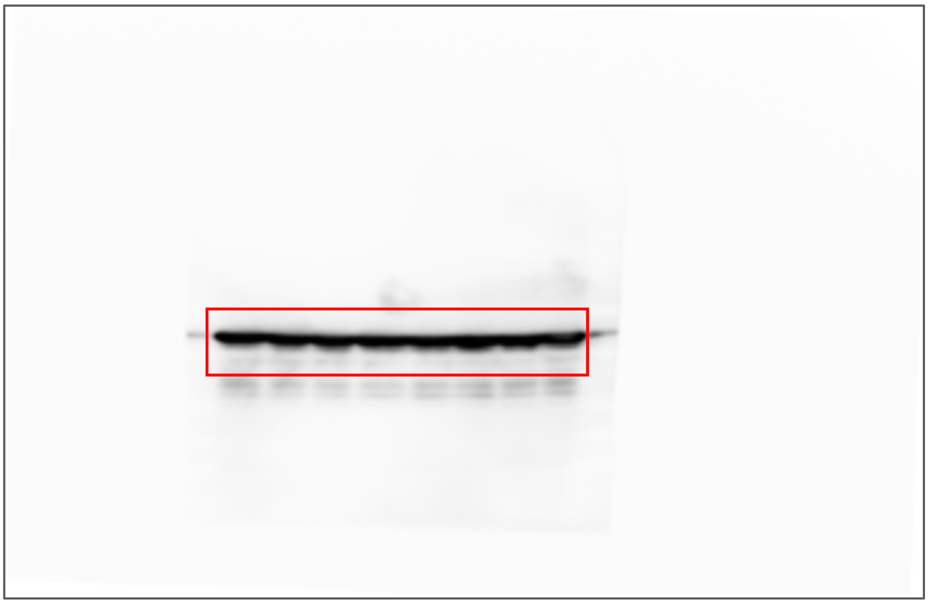

WB: ACTB (long exposure)

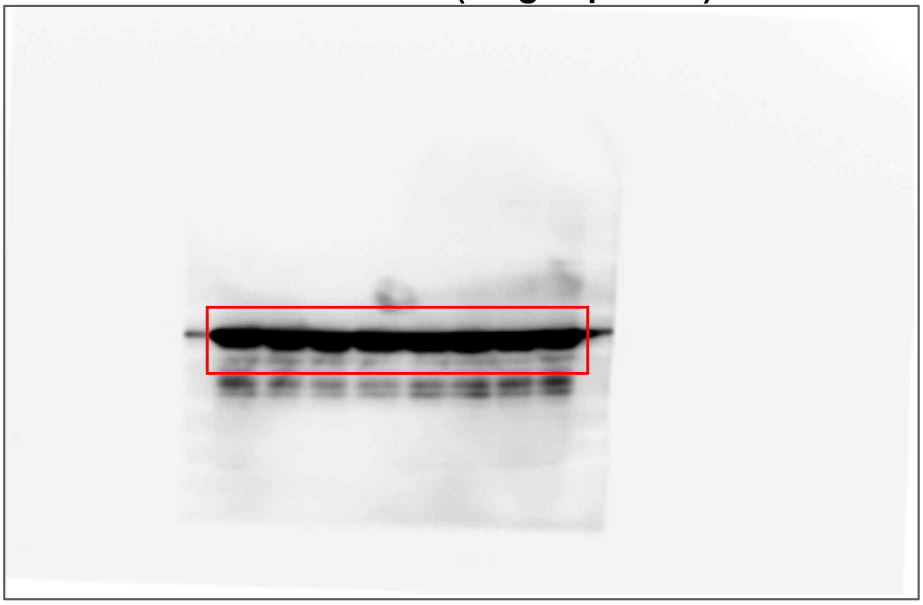

WB: ACTB (high contrast image of long exposure)

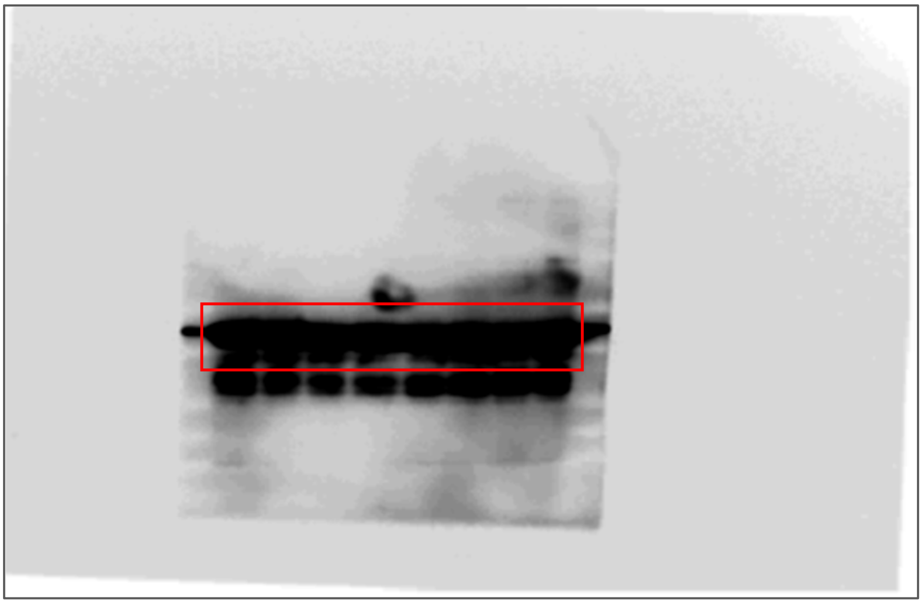

Supplement: Supplementary file 1 — Supplementary Information. [file 41598_2024_57367_MOESM1_ESM.pdf]
